# The Anticancer Peptide TAT-RasGAP_317−326_ Exerts Broad Antimicrobial Activity

**DOI:** 10.3389/fmicb.2017.00994

**Published:** 2017-06-07

**Authors:** Mathieu Heulot, Nicolas Jacquier, Sébastien Aeby, Didier Le Roy, Thierry Roger, Evgeniya Trofimenko, David Barras, Gilbert Greub, Christian Widmann

**Affiliations:** ^1^Department of Physiology, University of LausanneLausanne, Switzerland; ^2^Department of Laboratories, Institute of Microbiology, Lausanne University Hospital and University of LausanneLausanne, Switzerland; ^3^Infectious Diseases Service, Lausanne University HospitalEpalinges, Switzerland; ^4^Bioinformatics Core Facility, Swiss Institute of BioinformaticsLausanne, Switzerland

**Keywords:** TAT-RasGAP_317−326_, RasGAP, cell-permeable peptides, antimicrobial peptides

## Abstract

Antibiotic resistance has become a major health issue. Nosocomial infections and the prevalence of resistant pathogenic bacterial strains are rising steadily. Therefore, there is an urgent need to develop new classes of antibiotics effective on multi-resistant nosocomial pathogenic bacteria. We have previously shown that a cell-permeable peptide derived from the p120 Ras GTPase-activating protein (RasGAP), called TAT-RasGAP_317−326_, induces cancer cell death, inhibits metastatic progression, and sensitizes tumor cells to various anti-cancer treatments *in vitro* and *in vivo*. We here report that TAT-RasGAP_317−326_ also possesses antimicrobial activity. *In vitro*, TAT-RasGAP_317−326_, but not mutated or truncated forms of the peptide, efficiently killed a series of bacteria including *Escherichia coli, Acinetobacter baumannii, Staphylococcus aureus*, and *Pseudomonas aeruginosa*. *In vivo* experiments revealed that TAT-RasGAP_317−326_ protects mice from lethal *E. coli*-induced peritonitis if administrated locally at the onset of infection. However, the protective effect was lost when treatment was delayed, likely due to rapid clearance and inadequate biodistribution of the peptide. Peptide modifications might overcome these shortcomings to increase the *in vivo* efficacy of the compound in the context of the currently limited antimicrobial options.

## Introduction

A first line of defense provided by the innate immune system of multicellular organisms relies on the production of antimicrobial peptides. Since their initial discovery in the 1980s (Chan et al., 2006), over 2,000 antimicrobial peptides have been isolated from virtually all classes of living species, including humans, insects, plants, and bacteria themselves. Of interest, the peptide gramicidin, isolated from *Bacillus brevis* by René Dubos in 1939, was the first antibiotic to be commercially manufactured (Nakatsuji and Gallo, 2012). Usually, antimicrobial peptides are composed of 10–50 amino-acid residues and classified into different categories based on their amino-acid composition, size, and conformation (Nakatsuji and Gallo, 2012). They lack any obvious specific consensus amino-acid sequences associated with biological activity; yet most of them maintain certain common features, such as the presence of positively charged amino-acids or amphipathic nature. A majority of antimicrobial peptides interact with the bacterial membrane, causing defects in membrane integrity and ultimately inducing bacterial death (Chan et al., 2006; Nakatsuji and Gallo, 2012). Various models have been proposed to explain how given anti-bacterial peptides negatively impact on bacterial membrane integrity, by the formation of pores for example, but most of these models remain to be experimentally validated (Chan et al., 2006). It is now recognized that antimicrobial peptides can compromise bacterial viability independently of their action on membrane permeability, inhibiting for example protein or cell wall synthesis (Guilhelmelli et al., 2013). Rather surprisingly, despite their well-documented anti-bacterial properties, antimicrobial peptides have poorly attracted the interest of antibiotic producers that have rather focused on the development of small synthetic anti-bacterial molecules. With the emergence of antibiotic resistance against small antibiotic molecules and the steady decline in the discovery and release of new antibiotics, antimicrobial peptides hold the potential to provide an alternative source of potent antimicrobial agents (Chan et al., 2006).

TAT-RasGAP_317−326_ is a peptide composed of a cell permeable moiety, the TAT HIV 48–57 sequence, and a 10 amino-acid sequence derived from the Src Homology 3 Domain (SH3 domain) of p120 RasGAP (Michod et al., 2004). This peptide has various anticancer properties. It can sensitize tumor cells, but not normal cells, to anticancer treatments, such as chemotherapy, photodynamic therapy, and radiotherapy (Pittet et al., 2007; Michod et al., 2009; Tsoutsou et al., 2017). It has anti-metastatic properties by inhibiting cell migration and invasion (Barras et al., 2013, 2014a). TAT-RasGAP_317−326_ also directly kills a subset of cancer cells independently from apoptosis, necroptosis, and other forms of regulated death (Heulot et al., 2016). In this study, we uncovered an antimicrobial effect of TAT-RasGAP_317−326_. We show that this peptide can efficiently kill a broad spectrum of bacterial species *in vitro*. This peptide also confers efficient protection in a mouse model of peritonitis caused by *Escherichia coli* when administrated at the onset of infection.

## Results

### TAT-RasGAP_317−326_ possesses antimicrobial activities

**Figure 1:**
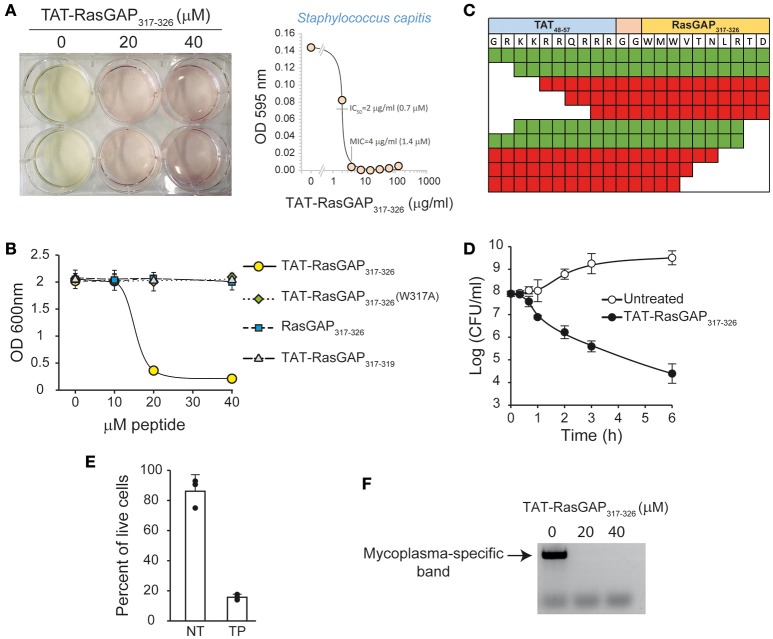
TAT-RasGAP_317−326_, but not mutated or truncated forms, has antimicrobial activities. **(A)** Contaminated U2OS cell cultures treated or not with 20 μM TAT-RasGAP_317−326_. Please observe the yellow color due to lower pH in presence of the growing contaminant. The contaminant was identified as *Staphylococcus capitis*. The panel on the right shows the sensitivity of this isolate to the peptide. The bacteria were grown in the presence of the indicated concentrations of TAT-RasGAP_317−326_. The OD 595 nm was measured after 16 h of incubation at 37°C. IC_50_, fifty percent maximal inhibitory concentration; MIC, Minimal inhibitory concentration. **(B)** *E. coli* DH5α [optical density (OD) 600 nm of 0.25] were incubated for 7 h at 37°C with the indicated concentrations of WT, mutated (W317A), or truncated TAT-RasGAP_317−326_ (RasGAP_317−326_ and TAT-RasGAP_317−319_) peptides. The OD 600 nm was then measured. The results correspond to the mean ± 95% CI of three independent experiments. **(C)** *E. coli* DH5α (OD 600 nm of 0.25) were treated with 30 μM of different truncated versions of TAT-RasGAP_317−326_ for 7 h at 37°C at which time bacterial density was recorded (OD 600 nm). White box represents a missing residue. Sequences highlighted in green allow at least 80% decrease of OD 600 nm compared to untreated condition. Sequences highlighted in red do not inhibit *E. coli* growth. The results are derived from three independent experiments. **(D)** *E. coli* DH5α (OD 600 nm of 0.25) were treated or not with 20 μM of TAT-RasGAP_317−326_ for the indicated periods of time at 37°C. Bacteria were diluted in bacterial culture medium without peptide and colony forming units (CFUs) were determined on agar plates. The results correspond to the mean ± 95% CI of three independent experiments. **(E)** *E. coli* DH5α (OD 600 nm of 0.25) were treated or not (NT) with 20 μM of TAT-RasGAP_317−326_ (TP) for 6 h at 37°C. The percent of live cells was determined using LIVE/DEAD kit. The results correspond to the mean ± 95% CI of three independent experiments. **(F)** U2OS cells were incubated 3 days with the supernatant of a *Mycoplasma hyorhinis*-infected cell culture. Then, 30,000 cells were plated in a 6-well plate and 24 h later treated with 0, 20, and 40 μM of TAT-RasGAP_317−326_ for 3 additional days. The medium was then replaced with fresh peptide-containing medium and the cells incubated for 10 more days. The supernatant of the culture was used to detect the presence of mycoplasma DNA by PCR as previously described (Uphoff and Drexler, 2005).

During an episode of contamination of mammalian cell cultures, we observed that the growth of an initially uncharacterized microorganism (later identified by sequencing as *Staphylococcus capitis*) was prevented when the culture medium contained the TAT-RasGAP_317−326_ peptide (Figure 1A). To extend this observation, *E. coli* DH5α were incubated with wild-type (WT), mutated (W317A), or a version of the peptide lacking the TAT cell permeable sequence. The W317A mutant peptide is known to be devoid of killing activity on eukaryotic cells (Heulot et al., 2016). As shown in Figure 1B, TAT-RasGAP_317−326_, but not the mutated or the TAT-deleted forms, prevented bacterial growth. Several additional N-terminal and C-terminal truncated versions of TAT-RasGAP_317−326_ were then tested. Figure 1C shows that removing up to two residues from either the N-terminus or the C-terminus did not abrogate the antimicrobial property of the peptides. In contrast, the antimicrobial activity was lost when four amino-acids or more were removed from either the N- or the C-terminus.

To determine if the peptide has a bactericidal or bacteriostatic effect, colony formation assays were performed. This allowed estimating the number of remaining viable and proliferation-proficient bacteria after peptide treatment. Figure 1D shows that the ability of *E. coli* DH5α to form colonies was reduced by more than one thousand fold after treatment with the peptide for 6 h. Moreover, bacterial membrane integrity, assessed by SYTO 9/propidium iodide staining, was greatly reduced after 6 h of peptide treatment, supporting the notion that TAT-RasGAP_317−326_ is bactericidal (Figure 1E).

To determine if the peptide targets intracellular bacteria, we tested its effect on a mammalian cell line contaminated with *Mycoplasma hyorhinis*. Mycoplasma are eukaryote commensals and can cause severe pathologies in humans (Uphoff and Drexler, 2005; Myers et al., 2010; Pascual et al., 2010). As shown in Figure 1F, the peptide efficiently cleared mycoplasma from infected eukaryote cell cultures.

### TAT-RasGAP_317−326_-mediated growth inhibition of potentially pathogenic bacteria

**Figure 2:**
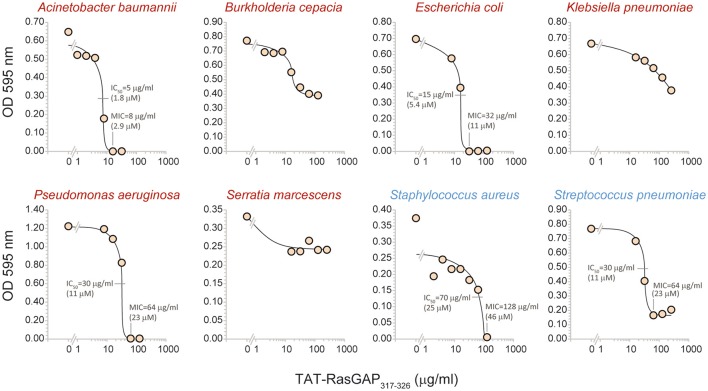
TAT-RasGAP_317−326_-mediated growth inhibition of ATCC bacteria. The indicated bacteria strains obtained from ATCC were grown in appropriate media in the presence of the indicated concentrations of TAT-RasGAP_317−326_. The OD 595 nm was measured after 16 h of incubation at 37°C. IC_50_, fifty percent maximal inhibitory concentration; MIC, Minimal inhibitory concentration.

**Figure 3:**
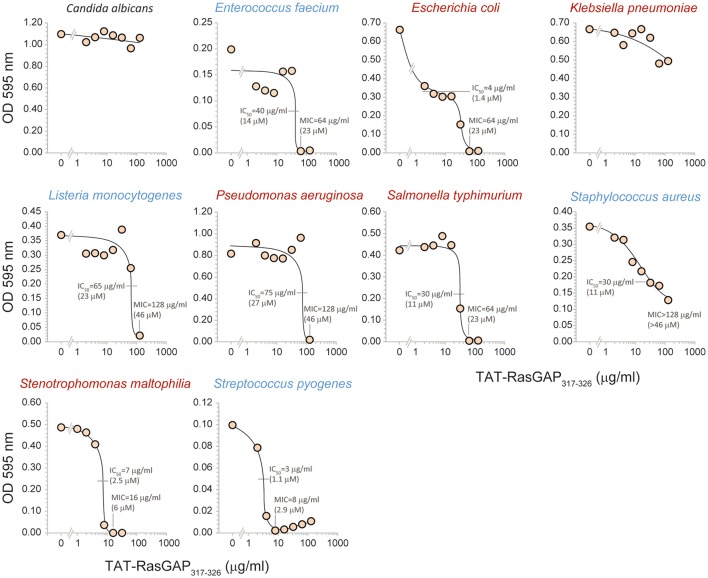
TAT-RasGAP_317−326_-mediated growth inhibition of pathogenic clinical strains. The indicated bacteria isolates were grown in presence of the indicated concentrations of TAT-RasGAP_317−326_. The OD 595 nm was measured after 16 h of incubation at 37°C. IC_50_, fifty percent maximal inhibitory concentration; MIC, Minimal inhibitory concentration.

**Figure 4:**
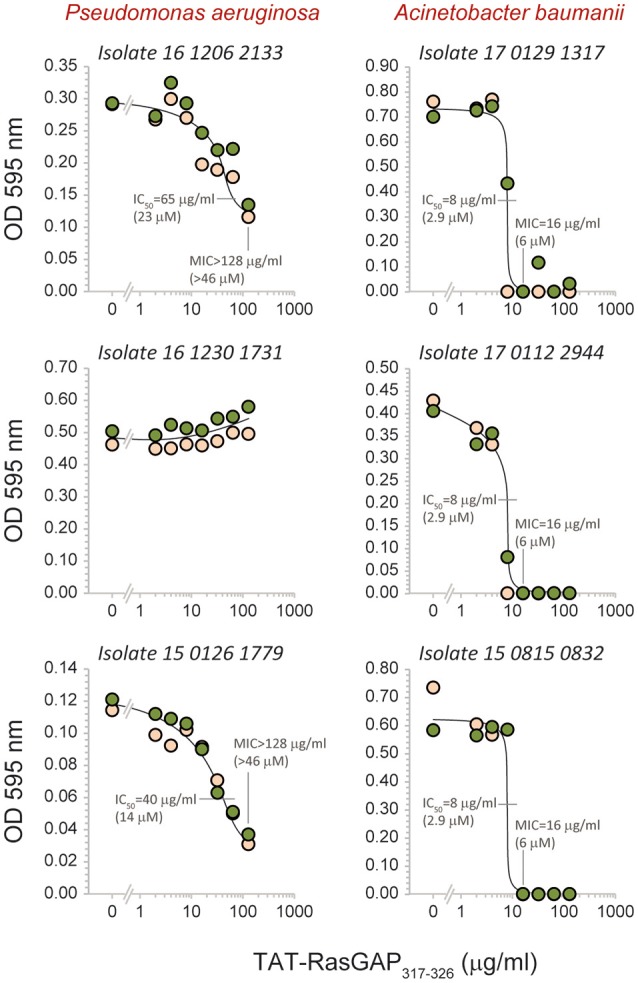
Effect of TAT-RasGAP_317−326_ on multi-drug resistant *Acinetobacter baumannii* and *Pseudomonas aeruginosa*. The indicated isolates were grown in the presence of the indicated concentrations of TAT-RasGAP_317−326_. The OD 595 nm was measured after 16 h of incubation at 37°C. IC_50_, fifty percent maximal inhibitory concentration; MIC, Minimal inhibitory concentration. The results were derived from two different experiments (the data from each experiment labeled with a different color).

To evaluate the spectrum of action of TAT-RasGAP_317−326_, its antimicrobial activity was assessed *in vitro* on several bacterial species that are potentially harmful to humans (listed in Table 1; their antibiotic sensitivity reported in Table 2). Figure 2 reports data obtained with bacterial strains originating from the ATCC collection. It shows that the growth of *Acinetobacter baumannii, E. coli, Pseudomonas aeruginosa, Staphylococcus aureus*, and *Streptococcus pneumoniae* was reduced in the presence of the peptide. *Burkholderia cepacia, Klebsiella pneumoniae*, and *Serratia marcescens* were not, or only partially, affected by TAT-RasGAP_317−326_. We then investigated the activity of the peptide on clinical isolates of various microbes. Figure 3 shows that the RasGAP-derived peptide inhibited the growth of patient-derived *Enterococcus faecium, E. coli, Listeria monocytogenes, P. aeruginosa, Salmonella typhimurium, S. aureus, Stenotrophomonas maltophilia*, and *Streptococcus pyogenes*. The yeast *Candida albicans* and the bacteria *K. pneumoniae* were not or poorly affected by TAT-RasGAP_317−326_. To determine whether the peptide was effective on multi-resistant bacteria, three independent clinical isolates of *Acinetobacter baumanii* and *P. aeruginosa* resistant to several classes of antibiotics (Table 3) were tested (Figure 4). The growth of all isolates of *Acinetobacter baumanii* was efficiently blocked by the peptide, while the growth of only two isolates of *P. aeruginosa* was inhibited and with lesser potency.

**Table 1:** Strains used in this study.

**Strain**	**Used in**		**Notes**
*Escherichia coli* DH5α	Figure 1		
*Staphylococcus capitis*	Figure 1		Mammalian cell culture contaminant
		**ATCC number**	
*Acinetobacter baumannii*	Figure 2	19606	
*Burkholderia cepacia*	Figure 2	25416	Polymyxine-resistant
*Escherichia coli*	Figure 2	25922	
*Klebsiella pneumoniae*	Figure 2	27736	
*Pseudomonas aeruginosa*	Figure 2	27853	
*Serratia marcescens*	Figure 2	8100	Polymyxine-resistant
*Staphylococcus aureus*	Figure 2	29213	
*Streptococcus pneumoniae*	Figure 2	49619	
		**Clinical isolate #**	
*Candida albicans*	Figure 3	5102	
*Enterococcus faecium*	Figure 3	2015 04201636	
*Escherichia coli*	Figures 3 and 5	O18:K1:H7	
*Klebsiella pneumoniae*	Figure 3	Caroli	
*Listeria monocytogenes*	Figure 3	10403s	
*Pseudomonas aeruginosa*	Figure 3	547	
*Salmonella typhimurium*	Figure 3	C5	
*Staphylococcus aureus*	Figure 3	7AW	
*Stenotrophomonas maltophilia*	Figure 3	2015 01100914	
*Streptococcus pyogenes*	Figure 3	H305	
*Acinetobacter baumanii*	Figure 4	3 different isolates	Antibiotic
*Pseudomonas aeruginosa*	Figure 4	3 different isolates	resistance profile shown in Table 3

*All strains were cultured in Cation Adjusted Mueller-Hinton medium. In the case of S. pneumoniae, this medium was complemented with 2.5% lysed horse blood. The strains are listed alphabetically and according to their use in the figures*.

**Table 2:** Antibiotic sensitivity of *Staphylococcus capitis* and strains used in Figures 2, 3.

**Strains**	**Amikacin**	**Amoxicillin-clavulanic acid**	**Ampicillin**	**Aztreonam**	**Benzylpenicillin**	**Cefepime**	**Cefotaxime**	**Cefoxitine**	**Ceftazidime**	**Ceftazidime-avibactam**	**Ceftlozane-Tazobactam**	**Ceftriaxone**	**Cefuroxime**	**Cefuroxime axetil**	**Ciprofloxacin**	**Clindamycin**	**Colistin**	**Co-trimoxazole**	**Daptomycin**	**Ertapenem**	**Erythromycin**	**Fosfomycin**	**Fusidic acid**	**Gentamicin**	**Imipenem**	**Levofloxacin**	**Linezolid**	**Meropenem**	**Minocyclin**	**Mupirocin**	**Nitrofurantoïne**	**Oxacillin**	**Piperacillin**	**Piperacillin-tazobactam**	**Rifampicin**	**Streptomycin**	**Teicoplanin**	**Tetracyclin**	**Ticarcillin-Clavulanate**	**Tigecyclin**	**Tobramycin**	**Trimethoprim-sulfamethoxazole**	**Vancomycin**
*Staphylococcus capitis*															S	S			S		S	R	S	S			S			S		S			S			S		S			S
*Acinetobacter baumannii*	S														S		S							R	S	S		S													S	R	
*Burkholderia cepacia*									S									S								I		I	S														
*Escherichia coli*	S	S	S			S		S	S			S	S	S	S					S		S		S	S			S			S			S								S	
*Klebsiella pneumoniae*	S	S	R			S		S	S			S	S	S	S					S		S		S	S			S			S			S								S	
*Pseudomonas aeruginosa*	S					S			S						S									S	S	S		S					S	S							S		
*Serratia marcescens*	S	R	R			S		R	S			R	R	R	S					S		S		S	S			S			R			I								S	
*Staphylococcus aureus*					R										S	S			S		S	S	S	S			S			S		S			S		S	S		S			S
*Streptococcus pneumoniae*			S		R							S				S					S					S												S				S	S
*Enterococcus faecium* (2015 04201636)		S	S															I						S	S								S			S	S						S
*Escherichia coli* (O18:K1:H7)	S	S	S			S		S	S			S	S	S	S					S		S		S	S			S			S			S								S	
*Klebsiella pneumoniae* (Caroli)	S	S	R			S		S	S			S	S	S	S					S		S		S	S			S			S			S								S	
*Pseudomonas aeruginosa* (547)	S					S			S						S									S	S	S		S						S							S		
*Staphylococcus aureus* (7AW)					R										S	R			S		R	S	S	R			S			S		S			S		S	R		S			S
*Stenotrophomonas maltophilia* (2015 01100914)																		S								S			S														

*Empty cells indicate that the given antibiotic was not tested. I (yellow), partially resistant; R (orange), fully resistant; S (green), sensitive. Antibiotic sensitivity was determined using the Vitek 2 apparatus (Biomérieux, Marcy-l'Etoile, France) for all strains except Burkholderia and Stenotrophomonas, the antibiotic sensitivity of which was tested using Agar diffusion tests with Kirby-Bauer medium. The background colors used in the “Strains” column refer to the grouping shown in Table 1*.

**Table 3:** Antibiotic sensitivity of clinical isolates of *Acinetobacter baumanii* and *Pseudomonas aeruginosa* (used in Figure 4).

**Species**	**Isolate number**	**Ticarcillin-Clavulanate**	**Piperacillin-tazobactam**	**Ceftazidime**	**Ceftazidime-avibactam**	**Cefepime**	**Aztreonam**	**Ceftlozane-Tazobactam**	**Imipenem**	**Meropenem**	**Amikacin**	**Gentamicin**	**Tobramycin**	**Colistin**	**Co-trimoxazole**	**Ciprofloxacin**	**Levofloxacin**
*Acinetobacter baumannii*	17 0129 1317								R	R	S	R	S	S	R	R	R
	17 0112 2944								R	R	S	R	R		R	R	R
	15 0815 0832								R	R	R	S	R	S	R	R	R
*Pseudomonas aeruginosa*	16 1206 2133		R	R		R			R	R	R	R	S	S		R	R
	16 1230 1731		R	R	R	R		R	R	R	R	R	S	S		R	R
	15 0126 1779	R	R	R		R	I		I	S	R	R	R	R		I	R

*Empty cells indicate that the given antibiotic was not tested. I (yellow), partially resistant; R (orange), fully resistant; S (green), sensitive. Antibiotic sensitivity was determined using the Vitek 2 apparatus (Biomérieux, Marcy-l'Etoile, France)*.

These data demonstrate that TAT-RasGAP_317−326_ has the ability to target both pathogenic Gram-positive and Gram-negative bacteria *in vitro*, some of which with pronounced resistance to multiple antibiotics used in the clinic. Of note, *B. cepacia* and *S. marcescens*, known to be naturally resistant to antimicrobial peptides, as well as to polymixins (Olaitan et al., 2014), an antibiotic class used as the last treatment option to disrupt both the outer and inner membranes of Gram-negative organisms (Yuan and Tam, 2008), were found also resistant, at least partially, to the peptide (Figure 2).

### TAT-RasGAP_317−326_ neither alters mouse health nor triggers hemolysis

**Figure 5:**
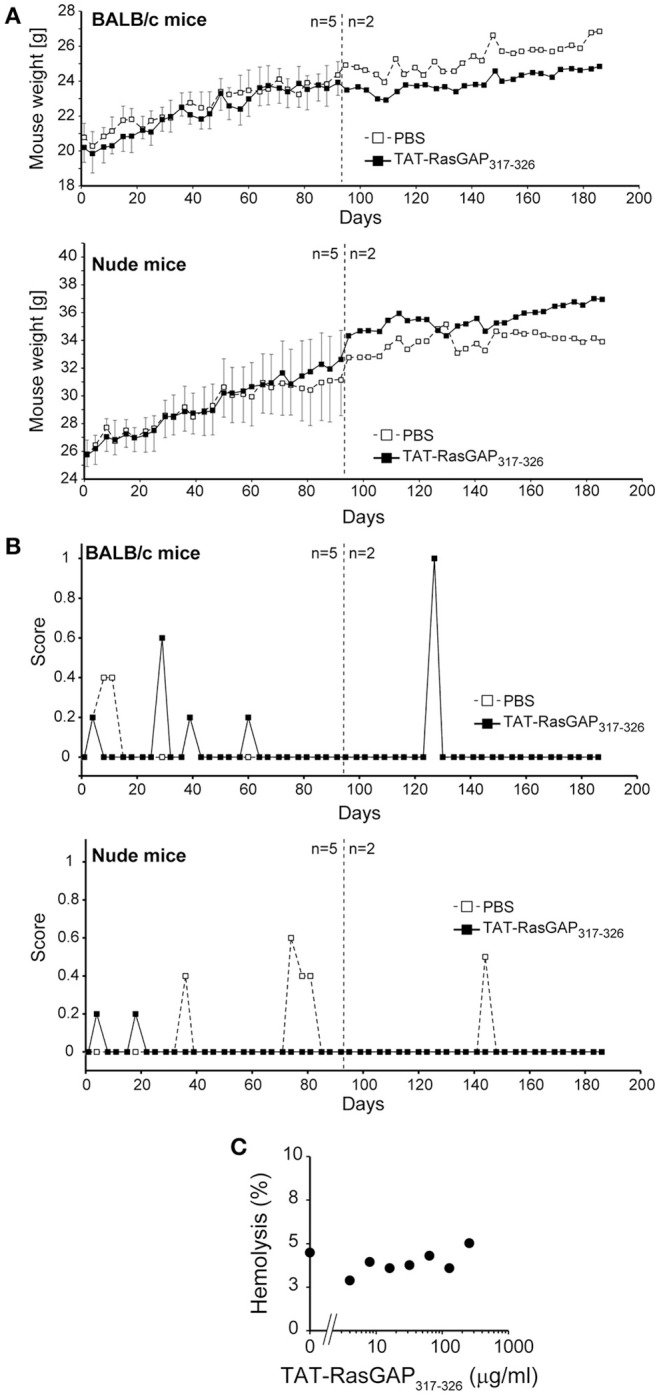
TAT-RasGAP_317−326_ does not alter mice health and does not trigger hemolysis. The indicated number of 8-week-old BALB/c and NMRI nude mice were i.p. injected twice a week with PBS or 1.6 mg/kg TAT-RasGAP_317−326_ (in PBS), weighed and evaluated for behavior and general aspect. After 90 days, three mice per group were sacrificed for organ inspection **(A)** Weight. The results correspond to the mean ± 95% CI. **(B)** General aspect and behavior score (see Section Materials and Methods). A score of 8 represents the limit above which mice need to be culled. **(C)** Hemolysis activity was determined by treating human red blood cells with various concentrations of TAT-RasGAP_317−326_. After 30 min incubation at 37°C, sample tubes were centrifuged and the absorbance of the supernatant was measured at 540 nm. Maximum hemolysis (set at 100%) was triggered with 0.5 % triton-X treatment of erythrocytes.

If TAT-RasGAP_317−326_ is to be used clinically, it has to display minimal cytotoxic activity *in vivo*. To address that point, a third of the mouse lethal peptide dose was injected twice a week for up to 6 months in BALB/c and NMRI nude mice (Michod et al., 2009). This treatment did not induce any deleterious effect or signs of distress in mice and it did not affect their growth and weight gain (Figures 5A,B). Moreover, inspection of organ sections (heart, lung, kidney, liver, thymus, spleen, pancreas, salivary gland, brain, stomach, small intestine, colon, uterus, ovary, fallopian tubes, tongue, skin, eyes, spine, and femur) did not reveal any difference between control and peptide injected groups. Additionally, the peptide displayed no hemolytic activity even at the highest dose tested (256 μg/ml ≈ 90 μM; Figure 5C). Overall, TAT-RasGAP_317−326_ seems not to display major adverse effects *in vivo*. There is therefore a potential therapeutic window for its use as an antimicrobial agent.

### TAT-RasGAP_317−326_ can protect from *E. coli* peritonitis

**Figure 6:**
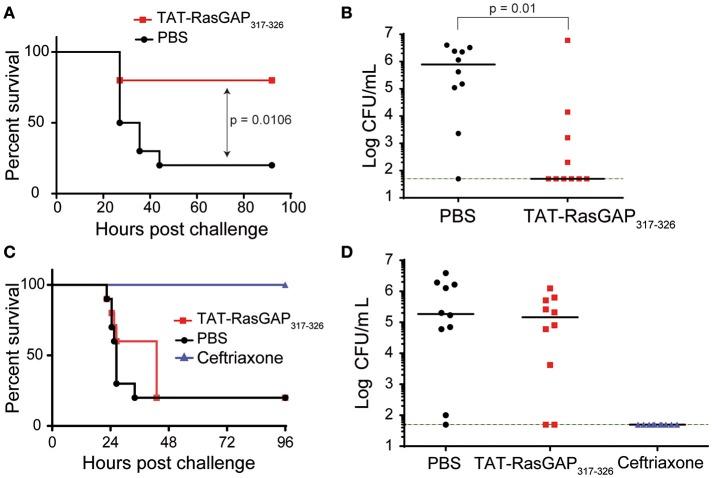
TAT-RasGAP_317−326_ can protect from *E. coli* peritonitis. **(A–D)** BALB/c mice (10 per group) were injected i.p. with 1.1 × 10^5^ CFU *E. coli* O18 and 2 min **(A,B)** or 2 h later **(C,D)** with PBS, 1 mg/kg TAT-RasGAP_317−326_ or 4 mg/kg ceftriaxone. **(A,C)** Survival of mice. **(B,D)** Bacterial counts in the blood 24 h post-infection. The green dashed line indicates the limit of detection.

Finally, we explored the antimicrobial and protective capacity of TAT-RasGAP_317−326_ in a mouse model of lethal peritonitis induced by *E. coli* O18 (Roger et al., 2009). Intraperitoneal (i.p.) administration of 1 mg/kg TAT-RasGAP_317−326_ 2 min after an i.p. injection of *E. coli* significantly increased survival rate from 20% (PBS control group) to 80% (Figure 6A), and markedly reduced the number of circulating bacteria 24 h post-infection (Figure 6B). The survival benefit was lost when the peptide was injected 2 h after *E. coli* (Figure 6C). In these conditions, no decrease in circulating bacteria was observed (Figure 6D). In contrast, mice treated with ceftriaxone (a broad-spectrum cephalosporin antibiotic) 2 h post-infection survived *E. coli* peritonitis and had no detectable bacteria in their blood. Taken together, these results demonstrate that, even if TAT-RasGAP_317−326_ possesses a broad-spectrum antimicrobial activity *in vitro*, its efficacy *in vivo* might be limited by factors such as inefficient biodistribution and rapid clearance (Michod et al., 2009).

## Discussion

Infections due to multi-resistant Gram-negative bacteria encoding extended spectrum beta-lactamase (ESBLs) and carbapenemases or Gram-positive bacteria such as methicillin resistant *S. aureus* and vancomycin-resistant enterococci among others, represent a very important public health challenge. The emergence of drug-resistant bacteria is expected to be only marginally prevented by active infection control (Kaspar et al., 2015; Morris et al., 2016). Thus, there is an urgent need for new antimicrobial agents to treat the increasing numbers of patients suffering from life-threatening infections due to multi-drug resistant Gram-negative and Gram-positive bacteria. Novel active antimicrobial compounds can be obtained from various sources including uncultured bacteria (Ling et al., 2015), human microbiota (Zipperer et al., 2016) or plant extracts (Tiwari et al., 2015, 2016) or they can be derived from known proteins such as the TAT-RasGAP_317−326_ peptide used in this study. We have uncovered in the present work that this peptide, previously shown to bear various anticancer properties, is an efficient antimicrobial agent toward a variety of pathogenic bacterial species *in vitro* (Figures 2–4). This substantiates a series of studies demonstrating that natural and synthetic antimicrobial peptides exhibit a broad spectrum of cytotoxic activity against cancer cells (Hoskin and Ramamoorthy, 2008; Riedl et al., 2011; Gaspar et al., 2013). Peptides with dual antimicrobial and anticancer activities often act via the disruption of bacterial and cancer cell membranes, such as the bovine BMAP-28 peptide (Risso et al., 2002) and melittin from bee venom (van den Bogaart et al., 2008). Their mode of action is generally not well-characterized and, in some cases, debated (Brogden, 2005). Lactoferricin B, for instance, was first reported to exert its antimicrobial and antitumoral activities via a pore-forming mechanism (Hwang et al., 1998; Eliassen et al., 2006). Other reports mention that lactoferricin B cytotoxicity is due to intracellular signaling perturbation in microbial and mammalian cells (Mader et al., 2005; Tu et al., 2011). We recently demonstrated that TAT-RasGAP_317−326_ kills some cancer cells in a caspase-, apoptosis-, and necroptosis-independent manner (Heulot et al., 2016). This raises the possibility that TAT-RasGAP_317−326_ exerts a lytic activity against certain cancer cells, potentially via membrane pore formation. TAT-RasGAP_317−326_, as many antimicrobial peptides, contains arginine and tryptophan residues (Reddy et al., 2004; Chan et al., 2006). Here, we show that substituting the tryptophan at position 317 by alanine completely abrogates the antimicrobial activity of the peptide *in vitro* (Figure 1B). Noteworthy, this key residue was previously reported to be crucial for all anticancer activities of the peptide (Barras et al., 2014b; Heulot et al., 2016). To date, the mode of action of TAT-RasGAP_317−326_ as an antimicrobial agent remains unknown.

**Figure 7:**
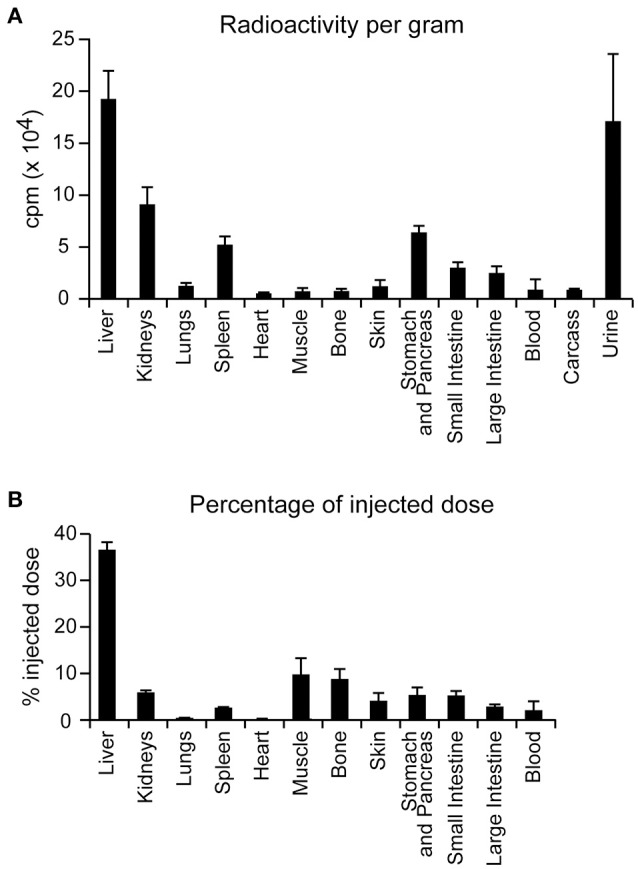
Biodistribution of I^125^-radiolabeled TAT-RasGAP_317−326_ in BALB/c mice. Five BALB/c mice were injected with 1.6 mg/kg I^125^-radiolabeled TAT-RasGAP_317−326_ peptide. Six hours after, mice were sacrificed and radioactivity of each organ mentioned in the figure was recorded in counts per minute (cpm). **(A)** Radioactivity of each organ normalized per gram of organ. Carcass was all the remaining mouse material after organ removal. Error bars are displayed in 95 percent confidence interval. **(B)** Percentage of the injected radioactive dose recorded in each organ. Error bars are displayed in 95 percent confidence interval.

TAT-RasGAP_317−326_ protected against *E. coli*-induced peritonitis when administered at the onset of infection. A 2-h delayed peptide injection did not prevent the spread of infection. This could potentially be due to inadequate peptide bio-distribution. Previous data reported that 1 h after i.p. injection, there is a peak of TAT-RasGAP_317−326_ concentration (of about 1 μM) in the blood that reached undetectable levels 2 h after injection (Michod et al., 2009). In addition, data obtained 6 h after i.p. injection with a radiolabeled version of the peptide revealed a preferential accumulation within the liver, kidneys, stomach and pancreas and a high concentration in urines, suggesting that the peptide is efficiently eliminated through the kidneys (Figure 7). Possibly, the TAT-RasGAP_317−326_ peptide could have a better impact on *E. coli* cystitis and/or pyelonephritis than on *E. coli* peritonitis. This could be tested in the future using previously reported pyelonephritis rat models (Glauser and Bonard, 1982). Presumably due to its suboptimal biodistribution and rapid clearance, the native version of the TAT-RasGAP_317−326_ peptide exhibits an efficient antimicrobial activity only within a very narrow time window. Biochemical modifications that improve its distribution and maintenance in whole organisms might therefore enhance its *in vivo* applicability. Finally, deciphering its mode of action might help identify new candidate molecules in bacteria that can be targeted for the development of novel potent antimicrobial agents.

## Materials and methods

### Bacteria

The bacteria strains used in this study are described in Table 1.

### Peptides

TAT-RasGAP_317−326_ is a retro-inverso peptide (i.e., synthesized with D-amino-acids in the opposite direction compared to the natural sequence). The TAT moiety corresponds to amino-acids 48–57 of the HIV TAT protein (RRRQRRKKRG) and the RasGAP_317−326_ moiety corresponds to amino-acids 317–326 of the human RasGAP protein (DTRLNTVWMW). These two moieties are separated by two glycine linker residues in the TAT-Ras-GAP_317−326_ peptide. TAT-RasGAP_317−326_ (W317A) has the tryptophan at position 317 mutated into an alanine. These peptides were synthesized at the department of biochemistry, University of Lausanne, Switzerland, using FMOC technology, purified by HPLC and tested by mass spectrometry.

### Cells and mycoplasma

The U2OS human osteosarcoma cell line (ATCC® HTB-96™) was cultured in DMEM (Invitrogen, ref. no. 61965) supplemented with 10% heat-inactivated fetal bovine serum (FBS; Invitrogen, ref. no. 10270-106) in 5% CO_2_ at 37°C. We obtained these cells from a laboratory that does not routinely screen for mycoplasma contamination. These cells were analyzed for the presence of mycoplasma-specific sequences. This was done by PCR amplification of supernatant of super confluent cell cultures using primers able to amplify *Mycoplasma, Acholeplasma, Ureaplasma*, and *Spiroplasma* (sense primer #519: GGG AGC AAA CAG GAT TAG ATA CCC T and anti-sense primer #520: TGC ACC ATC TGT CAC TCT GTT AAC CTC; van Kuppeveld et al., 1992). The obtained PCR product was then sequenced and found to correspond to *M. hyorhinis* sequences.

### Inhibition of bacterial growth

Bacterial growth was tested by optical density (OD) measurements. The laboratory strain DH5α transformed with pcDNA3 was cultured at 37°C in LB medium containing 100 μg/mL ampicillin. Bacteria were seeded at an OD 600 nm of 0.25 and treated with the indicated concentrations of peptides. OD 600 nm was measured after 7 h of incubation.

The clinically relevant strains were grown in cation-adjusted Mueller-Hinton medium. OD 595 nm was measured at 16 h of treatment with various concentrations of TAT-RasGAP_317−326_. IC50 and MIC were determined as described (Leber, 2016).

### Colony formation assay

*E. coli* DH5α transformed with pcDNA3 were cultured at 37°C in LB medium containing 100 μg/mL ampicillin. Bacteria were seeded at OD 600 nm of 0.25 and treated with indicated concentrations of peptide. After the indicated time periods, the bacterial suspension was diluted (from 10^−2^ to 10^−6^) in fresh medium and 100 μL plated on LB agar plate containing 100 μg/mL ampicillin. After overnight incubation at 37°C, colonies were counted.

### Bacterial viability

*E. coli* DH5α transformed with pcDNA3 were cultured at 37°C in LB medium containing 100 μg/mL ampicillin. Bacteria were seeded at OD 600 nm of 0.25 and treated or not with 20 μM TAT-RasGAP_317−326_ for 6 h. Bacterial viability was assessed with LIVE/DEAD BacLight Bacterial viability kit (Molecular Probes, ref. no. L7012) according to manufacturer's instructions using Cytation3 (BioTek, ref. no CYT3MV) as a microplate reader.

### Hemolytic activity

Hemolytic activity was measured as described in Ando et al. (2010). Briefly, blood (2 mL), derived from anonymous healthy human blood donors obtained through the Vaud blood transfusion service, were mixed with 2 mL PBS and centrifuged at 800 g for 5 min at 4°C. Samples were washed three times with PBS and resuspended in 2 mL PBS and further diluted with 18 mL PBS. This erythrocyte solution was diluted 10 times with PBS before treatment with various amounts of TAT-RasGAP_317−326_ peptide. After 30 min incubation at 37°C, samples were centrifuged at 800 g for 5 min at 4°C and the absorbance at 540 nm of the supernatant was measured. As a positive control (100% lysis), erythrocytes (200 μL of the final dilution preparation) were treated with 0.5% Triton X-100.

### *In vivo* toxicity assessment

Five-week-old NMRI nude and BALB/c mice (Charles River Laboratories) were injected i.p. with PBS only or with 1.6 mg/kg of TAT-RasGAP_317−326_ diluted in PBS every Monday and Thursday during 6 months. Body weight as well as behavioral and general aspect parameters (appearance, food and water intake, natural behavior, provoked behavior) were monitored according to FELASA recommendations (Table 4).

**Table 4:** Scoresheet used to assess the impact on the health and behavior of experimentally treated mice.

**Parameter**	**Animal ID**	**Score**
Appearance	Normal	0
	General lack of grooming	1
	Staring coat, ocular, and nasal discharge	2
	Piloerection, hunched up	3
Food and water intake	Normal	0
	Uncertain, body weight loss <5%	1
	Intake reduced: body weight loss 5–15%	2
	No food or water intake	3
Natural behavior	Normal	0
	Minor changes	1
	Less mobile and alert, isolated	2
	Vocalization, self-mutilation, restless or still	3
Provoked behavior	Normal	0
	Minor depression or exaggerated response	1
	Moderate change in expected behavior	2
	Reacts violently, or very weak or pre-comatose	3

### Mouse models of infections

BALB/cByJ mice (8–10 week-old females; Charles River Laboratories) were weighed and randomly distributed into groups of 9–10 animals of equal mean body weight. Mice were injected i.p. with 1.1 × 10^5^ CFU *E. coli* O18 (Ciarlo et al., 2016). Two minutes after bacterial challenge, mice were injected i.p. with PBS, TAT-RasGAP_317−326_ diluted in PBS or ceftriaxone as described in the figure legends. Mice were monitored at least twice daily to register severity scores, body weight, and survival as described (Roger et al., 2013). Blood samples were harvested from the facial vein for quantification of circulating bacteria. Survival curves were generated using the Kaplan-Meier method and differences were analyzed by the log-rank sum test. Statistical differences for bacterial blood counts were assessed using the non-parametric Mann–Whitney test. Analyses were performed using PRISM (GraphPad Software). All reported *P*-values are two-sided and values of <0.05 were considered to indicate statistical significance.

## Ethics statement

All animal procedures were approved by the Service de la Consommation et des Affaires Vétérinaires du Canton de Vaud (authorization 877-8) and performed according to the institution and ARRIVE guidelines for animal experiments.

## Author contributions

Conception and design of study: MH, TR, GG, and CW. Acquisition of data: MH, NJ, SA, DL, and ET. Analysis and/or interpretation of data: MH, NJ, DL, TR, DB, GG, and CW. Drafting the manuscript: MH and CW. Revising the manuscript and approval of the submitted version: MH, NJ, SA, DL, TR, ET, DB, GG, and CW.

### Conflict of interest statement

The authors declare that the research was conducted in the absence of any commercial or financial relationships that could be construed as a potential conflict of interest. The reviewer OS and handling Editor declared their shared affiliation, and the handling Editor states that the process nevertheless met the standards of a fair and objective review.
